# National hospital quality measures for surgical site infections in South Korea: a survey among 20 expert physicians

**DOI:** 10.1186/s13037-020-00255-5

**Published:** 2020-07-13

**Authors:** Mikyung Ryu, Hyerim Yoo, Yun-Kyoung Choi

**Affiliations:** 1 College of Nursing and Public Health, Daegu University, 33 Seongdang-ro 50-gil, Nam-gu, Daegu, Seoul, 42400 South Korea; 2 Review & Assessment Research Department, Health Insurance Review & Assessment Service, 60 Hyeoksin-ro, Wonju-si, Seoul, Gangwon-do 26465 South Korea; 3 Department of Nursing, Korea National Open University, 86, Daehak-ro, Jongno-gu, Seoul, 03087 South Korea

**Keywords:** Quality indicators, Surgical wound infection, Physicians, Perception

## Abstract

**Background:**

Surgical site infection (SSI) is recognized as an important quality indicator for patient safety. In Korea, the use of prophylactic antibiotics for surgery is conducted as a national quality measures related to SSI prevention. The objective of the present study was to investigate physicians’ perceptions of hospital quality measures for SSI as well as identify obstacles that might hinder its implementation in South Korea.

**Method:**

Online-based questionnaires were administered twice. Twenty physician experts who were members of the Healthcare Review and Assessment Committee that was constituted for the “Assessment of prophylactic use of antibiotics for surgery” participated in the study. The first survey comprised open-ended questions that were designed to elicit the physician who could hinder the implementation of SSI indicators. The second survey, which was developed on the basis of the initial survey’s results, consisted of 10 closed-ended questions about the feasibility of objective perception and the need for subjectivity, with regard to SSI.

**Results:**

From among the 20 physicians, we collected data from 16 respondents in the first survey (response rate of 80%) and 15 respondents in the second survey (response rate of 75%).Thirty-one percent of the respondents supported hospital SSI evaluations, and 69% expressed objections. The obstacles that were perceived as being able to hinder hospital SSI evaluations pertained to difficulties in collecting data, unavailability of information, possibility of underreporting, and redundancy of the inquiry undertaken by the Korean National Healthcare-associated Infections Surveillance System-SSI. Physician experts provide significantly higher ratings for the clinical indicator, rate of readmission due to SSI, both in terms of feasibility and need, when evaluating the results of SSI prevention in hospitals.

**Conclusion:**

The results of this study show that physicians perceive the need for QI development of hospital SSI measurements to prevent nation-wide SSIs in Korea. However, the feasibility of hospital SSI measurements is low. To develop QIs of hospital SSIs using health insurance claims data, it is necessary to develop a methodology for claims data-based surveillance systems and a data collection system in order to increase the sensitivity and validity of post-operative SSI detection.

## Background

Infections that are absent and not latent at the time of admission but emerge during the hospital stay are known as healthcare-associated infections (HAIs) [1], One such infection is the surgical site infection (SSI), which is the third-most common HAIs, following urinary tract infection and respiratory infection. SSI can result in prolonged hospital stays, readmission, additional medical and social costs, and an increased risk for mortality and morbidity [2–4]. However, about 60% of SSI can be prevented by adhering to evidence-based guidelines [5], thereby making SSI prevention a possibility within the quality improvement (QI) activities of healthcare organizations.

The World Health Organization reports that the incidence of SSI is 6.0% in low-income countries and 7.8% in Southeast Asian countries, both of which are higher than those of countries such as the United States and Australia where the incidence rates are 0.9 and 2.8%, respectively [6]. Further, a systematic review of Korean studies showed that the incidence of SSI in Korea ranges between 2 and 9.7% [7], and according to data from the Korean Nosocomial Infection Surveillance System (KONIS) between 2008 and 2012, the SSI rate following gastrectomy was 3.12%, that for total hip arthroplasty was 2.05%, and that for total knee arthroplasty was 1.90% [8], all of which are higher than the rates that have been recorded in other countries; therefore, there is a need for nationwide efforts to lower the incidence of SSI.

To decrease the likelihood of SSI, it is important to first identify risk factors before developing the necessary measures for its prevention and management. Most SSI are caused by normal skin flora at the surgical incision site. Therefore, the risk of infection is higher when the surgical site is open, from the time of skin incision until the wound is sutured. Other external causes of SSI include contaminated surgical environments and instruments [9]. Further, SSI can also be influenced by the patient’s underlying medical condition, immune status, surgical wound classification grades, and type of surgery [6].

The administration of prophylactic antibiotics is an effective strategy to lower the risk of SSI caused by normal skin flora. It also ensures an appropriate use of antibiotics, reduction of misuse or abuse of antibiotics, and prevention of multi-resistant bacteria. To this end, an appropriate selection of antibiotics based on the type of surgery, and measures to maintain permissible levels of antibiotics in blood and tissue during a surgery will be helpful. It is noteworthy that, since 2007, the Health Insurance Review and Assessment Service’s (HIRA) National Quality Assessment Program, which has been assessing the prophylactic use of antibiotics for surgeries in the prevention of SSI, has managed to decrease the abuse and misuse of antibiotics. As a result of this program, the administration of prophylactic antibiotics, within 1 h of surgical incision, improved from 70% (in the first assessment) to 88.2% (in the seventh assessment). Additionally, the administration of aminoglycoside, one of the antibiotics that should only be used sparingly recorded a decrease in its usage, dropping from 30 to 3% [10].

Meanwhile, it has been suggested that the assessment should measure the outcomes of QI activities in healthcare organizations in a manner that it is inclusive of the various factors that affect SSI. In other words, it should address, the limitations of current assessments, which are largely focused on the prophylactic use of antibiotics for surgery [11]. Although the lower incidence of SSI is a highly prioritized outcome indicator, its measurement, requires motivations on the part of the hospital in carrying out takes such as collecting relevant data, participating in assessments, and accepting assessment results. Therefore, the introduction of SSI assessment indicators for the prevention of SSI must be considered at this point. In this context, this study aims to investigate physicians’ perceptions of SSI assessment indicators, and the factors that are likely to hinder their implementation, to obtain basic data for the development of outcome indicators for assessing the prophylactic use of antibiotics in surgery.

## Method

### Participants

Twenty physician expert members, who were members of the Healthcare Benefits Review and Assessment Committee that was constituted for the ‘Assessment of prophylactic use of antibiotics in surgery’, were recruited as per the recommendations of each academic society. The HIRA sent official letters to academic societies to solicit recommendations from committee members with knowledge and expertise in the assessment of prophylactic use of antibiotics for surgery.

The expert panel included 13 surgeons as recommended by The Korean Surgical Society, The Korean Neurosurgical Society, The Korean Orthopedic Association, and the Korean Society for Thoracic and Cardiovascular Surgery. An additional 2 internists were recommended by The Korean Society of Infectious Diseases and The Korean Association of Internal Medicine, and 5 additional surgeons were recommended by The Korean Society of Otorhinolaryngology-Head and Neck Surgery, The Korean Society of Gynecologic Oncology, The Korean Ophthalmological Society, and The Korean Urological Association.

### Data collection

Two rounds of online surveys were carried out to investigate physicians’ perceptions of SSI assessment indicators and factors that could hinder its use. In the first-round survey, expert opinions were obtained by administering an open-ended questionnaire to physicians. In the second round, the opinions collected in the first round were used to design a closed-ended questionnaire, to investigate the survey objective and subjective perceptions. This study was approved by the Institutional Review Board (IRB No.2018–029) at the HIRA, after considering the ethical problems that may arise during the study.

### Instruments

Based on a consensus among the members of the department assessing the prophylactic use of antibiotics for surgery and experts in the first survey, the following contents about the SSI indicator assessment were administered as open-ended questions:

- “What is the possibility and limitations of assessing SSI incidence based on outcome indicators (obstacles to be overcome)?”
- “Which factors should be considered in developing items for quality measures for SSI?”
- “Which existing indicators, other than SSI, can that be used as outcome measures?”

In the second round, opinions collected from the first round were used to design 10 close-ended questions, and participants were asked to rate the feasibility and need, for each item on the of SSI indicator assessment. The feasibility of an SSI indicator is rated on a five-point scale, to measure physicians’ decisions and opinions, whereas the need for an SSI indicator assessment is rated on a four-point scale (excluding neutral opinions) to measure physicians’ perceptions irrespective of factual information.

### Data analysis

The frequency of responses on the Likert scale, ranging from 1 to 5, was converted to percentages (%), as shown in Table 1. Subsequently, these percentages were divided into three qualitative groups, as shown in Table 2 [12]. Responses on the subjective need for an SSI indicator assessment were analyzed with reference to previous studies, as shown in Table 3 [12].

**Table 1:** Feasibility percentages for the SSI assessment indicators

Likert scale	% value^a**)**^
1	96.00
2	73.25
3	50.50
4	27.75
5	5.00

Note. ^a^) % is calculated by y = (− 22.75) x + 118.5, if x is Likert scale, y is %

**Table 2:** Percentage-based feasibility classification for the SSI assessment indicators

Feasibility percentage (%)	Degree of feasibility
**Over 67**	High
**51 ~ 66**	Moderate
**50 and below**	Low

**Table 3:** Classification of subjective need for the SSI assessment indicators

Score of SSI indicator assessment (Conversion Value)	Interpretation
Lower than 2.00	Agree
2.01 ~ 2.90	Partially agree /Partially disagree
Over 2.91	Disagree

## Results

From July 30, 2018 to August 17, 2018, data were collected from 16 participants in the first survey (response rate of 80%) and 15 participants in the second survey (response rate of 75%). Table 4 shows the general characteristics of the physician expert panel that participated in the study.

**Table 4:** General characteristics of participants

Variables		Survey 1	Survey 2
		N (%)	
Total		16 (100)	15 (100)
Gender	Male	14 (87.5)	13 (86.7)
	Female	2 (12.5)	2 (13.3)
Age (years)	Mean (SD)	48.5 (5.57)	48.1 (5.48)
	40s	9 (56.3)	9 (60.0)
	50s	6 (37.5)	6 (40.0)
	60s	1 (6.3)	0
Experience (years)	Mean (SD)	21.13 (7.45)	20.67 (7.47)
	10 ~ 19	5 (31.3)	5 (33.3)
	20 ~ 29	9 (56.3)	8 (53.3)
	30 ~ 39	2 (12.5)	2 (13.3)
Department	Orthopedics	4 (25.0)	4 (26.7)
	General Surgery	3 (18.8)	3 (20.0)
	Infectious Disease	2 (12.5)	2 (13.3)
	Cardiothoracic Surgery	2 (12.5)	2 (13.3)
	Others ^a)^	5 (31.3)	4 (26.7)

Note. ^a^) Endocrine Surgery, Urology, Neurosurgery, Ophthalmology, Ear-nose-and-throat department

In the first open-ended survey, 31% of the participants (*n* = 5) were found to have positive opinions about the implementation of SSI indicators; the remaining 69% (*n* = 11) were found to have negative opinions. Participants perceived difficulties in obtaining accurate information, collecting data, possible underreporting, and redundant monitoring systems by the KONIS to be some of the factors that could hinder the implementation of SSI indicators. Further, rate of readmission due to SSI was suggested as an alternative indicator.

Table 5 shows the results that were derived from the responses that participants provided in the second 10 item survey, which was developed based on the findings of the first survey. Severity-adjusted SSI readmission rate was perceived to be a highly feasible assessment indicator, as was evidenced by the fact that most of the participants were in favor of it.

**Table 5:** Summary of physicians’ perceptions of national hospital quality measures for SSI, in terms of feasibility and need

Questions	Feasibility		Need	
	Conversion value	Interpretation	Conversion value	Interpretation
Is increasing the percentage of indicators related to patient safety?	64.2	Moderate	2.13	Partially agree/Partially disagree
Is increasing the percentage of outcome indicators in the “Assessment of prophylactic use of antibiotics for surgery”?	59.6	Moderate	2.07	Partially agree/Partially disagree
Do you accept that the severity is adjusted among SSI indicators so as to increase the possibility of comparison?	56.6	Moderate	2.33	Partially agree/Partially disagree
Can SSI indicators be developed based on the SSI items used in existing assessment checklists?	56.6	Moderate	2.20	Partially agree/Partially disagree
Can a checklist be developed to help patients voluntarily report SSI after discharge?	56.6	Moderate	2.40	Partially agree/Partially disagree
Can SSI incidence data be obtained by linking with the KONIS SSI monitoring system?	61.1	Moderate	2.20	Partially agree/Partially disagree
Can the SSI readmission rate be accepted if the severity is adjusted to increase the possibility of comparison among hospitals?	71.7	High	1.93	Agree
Should SSI readmission rate be developed by excluding relatively mild SSI (outpatient treatment) and focusing on serious adverse events requiring hospitalization?	64.2	Moderate	1.93	Agree
Should SSI readmission rates be accurately surveyed to check only cases involving readmission to the same hospital?	61.0	Moderate	1.93	Agree
Are indicators such as SSI prevention education, operating room environment, surgery preparation process, and standard prevention guidelines, more useful?	65.7	Moderate	1.93	Agree

Note. *SSI* Surgical Site Infection, *KONIS* Korea Nosocomial Infections Surveillance

## Discussion

Regarding the introduction of SSI assessment indicator in the evaluation of medical institutions, physicians perceived them to be important indicators that have implications for patient safety. However, they appraised the possibility of implementation and measurement of such indicators to be low, with the most significant obstacle being difficulties in collecting accurate SSI data. A review of the following issues is likely to point towards necessary measures that can aid with SSI data collection and assessment.

First, there needs to be a system for investigating infections that occur after discharge. In the survey on prophylactic use of antibiotics in surgery, variables are collected based on SSI definition, however the information is limited to that which is gathered during the hospital stay. According to the Centers for Disease Control and Prevention (CDC) definition, SSI includes cases that occur up to 30 days after surgery and up to 1 year after surgery, if artificial devices have been inserted into the human body [13]. On the other hand, KONIS’ SSI monitoring system defines SSI in such manner that includes cases that occur up to 90 days after the insertion of artificial substances into human body [14]. An American retrospective cohort study reported that SSI data are generally limited to infections that occur during hospital stay or those that lead to readmission to the same hospital. They further speculated that, if there were SSI cases that had been admitted to hospitals in which they did not undergo the respective surgery, the actual rate would be higher than the reported rate [15]. Supporting such a contention, Yokoe et al. [16] estimated SSI incidence by including cases in which the patient visits or is readmitted to another hospital and found that the rate varies across hospitals. Furthermore, because early discharge is encouraged after surgery and many surgeries do not require postoperative management at the same hospital after discharge, there is a demand for an alternative system to monitor SSI cases that occur after discharge.

Second, there is a need for efficient data collection measures that could increase the sensitivity and validity of SSI data. For instance, the assessment checklist for the prophylactic use of antibiotics in surgery is a self-reported survey of SSI events that occur in each hospital. Similarly, the KONIS, which was implemented in 2007, is an internet-based prospective surgical site monitoring system that collects data based on medical records. A limitation of this system is that SSI may be under-reported when relevant information is not entered in medical records [14]. Data are often obtained from health insurance claims rather than patient medical records because it is an efficient method of making the data immediately available to many people [17]. Indeed, detection of SSI cases, based on claims data that might have been omitted during routine surveillance, may be used as an initial screening technique, prior to the more rigorous investigation of medical records [18]. Calderwood et al. [19] assessed regularly collected electronic Medicare claims data to obtain postoperative SSI data. He developed an SSI indicator coding system for specific surgeries and found that SSI detection rate, based on claims data, was approximately 1.8–4.7 times higher. In another study that compared the validity of claims code-based SSI surveillance and cases reported by medical records, conventional surveillance method was found to have a low sensitivity of about 50–68% but high specificity of approximately 99.7% [20]. On the other hand, surveillance using claims data was found to have a higher sensitivity (74–84%) with a positive predictive value of 40–60%. Further, work efficiency was found to be approximately 2–2.6 times higher, when the number of patients reviewed per detection was considered [20].

Third, to develop a severity adjustment model, more studies are needed to investigate those SSI risk factors that are specific to the demographic features of Korea. This is significant because, in studies that examined hospital readmission and SSI predictors, the identified SSI risk factors were found to differ depending on whether American, Danish, or Japanese databases were used [21]. Although mediating factors and patient populations may have differed, these results suggest that risk stratification models developed in a specific region or country cannot be generalized to another country.

In consideration of the above limitations, there may be a few short-term strategies to measure SSI. First, the survey can be limited to types of surgeries that require the patient to visit the hospital for follow-up care after discharge. Follow-up is more feasible in the cases of surgeries in which patients are highly likely to visit the same hospital for post-surgical complications and infections. Second, additional codes should be developed, and computer systems should be updated to enable monitoring of SSI, based on health insurance claims data. Further, claims data and medical records data should be compared; once the validity of the data is established, they can be used as assessment indicators. Third, measures to integrate the KONIS-SSI system and health insurance claims data should be considered. As previously mentioned, detection of SSI that relies on integrated data derived from health insurance claims and detailed KONIS medical records is likely to create a more efficient and robust information collection system. Since January 2018, medical institutions are required to participate in KONIS, which is operated by the Korean CDC, to request for infection prevention and management fees. In 2019, this requirement will be expanded to hospitals; therefore, it is possible that SSI-related data can be interlinked across hospitals that participate in KONIS.

This study has a number of limitations. The first limitation is the small number of respondents, although they are experts recommended by academic societies. The second limitation is the use of the Likert scale. We used a 5-point scale in the first online survey and a 4-point scale for the second survey, as we decided to exclude neutral opinions. This, however, can be problematic in regard of coherence. Nevertheless, this study is the first attempt at the perceptions of SSI indicators and hindering factors. The study can provide basic data for the development of outcome indicators for national hospital quality measures for SSI.

## Conclusion

Even if both claims data and medical records data are used, it is difficult to resolve the issue of short-term follow-up of SSI after discharge. In our survey, more than two-thirds of the experts held negative opinions about the implementation of SSI indicators because of inaccurate data collection, underreporting, and overlapping data collection with KONIS. However, SSI readmission rates evidenced a higher score for physicians’ objective perception of feasibility and subjective need, when compared to other SSI indicators. Thus, SSI readmission indicator appears to be more practical and feasible in the short-term because relatively accurate information can readily be obtained from claims data and medical records. Furthermore, because readmission due to SSI is a serious adverse event in terms of patient safety, monitoring and management of this parameter by medical institutions are crucial.

Monitoring of SSI incidence and hospital-led QI activities must take precedence so that data about SSI indicators can be used to assess the quality of healthcare organizations and establish financial incentives based on performance.

## Data Availability

Not applicable.

## Abbreviations

SSI: Surgical Site Infection
HAIs: Healthcare-Associated Infections
HIRA: Health Insurance Review and Assessment Service
KCDC: Korea Centers for Disease Control and Prevention
KONIS: Korean Nosocomial Infections Surveillance
QI: Quality Improvement
